# Occurrence of Phthalates in Bottled Drinks in the Chinese Market and Its Implications for Dietary Exposure

**DOI:** 10.3390/molecules26196054

**Published:** 2021-10-06

**Authors:** Xiaohong Xue, Yaoming Su, Hailei Su, Dongping Fan, Hongliang Jia, Xiaoting Chu, Xiaoyang Song, Yuxian Liu, Feilong Li, Jingchuan Xue, Wenbin Liu

**Affiliations:** 1 College of Science, Dalian Maritime University, 1 Linghai Road, Dalian 116026, China; xuexiaohong@dlmu.edu.cn; 2 South China Institute of Environmental Sciences, Ministry of Ecology and Environment of P.R.C., No. 7, West Street, Yuancun, Tianhe District, Guangzhou 510535, China; suyaoming@scies.org (Y.S.); fandp0104@163.com (D.F.); 3 State Key Laboratory of Environmental Criteria and Risk Assessment, Chinese Research Academy of Environmental Sciences, Beijing 100012, China; su.hailei@craes.org.cn; 4 International Joint Research Centre for Persistent Toxic Substances (IJRC-PTS), College of Environmental Science and Engineering, Dalian Maritime University, Dalian 116026, China; jiahl@dlmu.edu.cn (H.J.); liuxuesong1998@163.com (X.C.); 5 Dalian Modern Agricultural Production Development Service Center, 678 Zhongshan Road, Dalian 116026, China; sun_7812@163.com; 6 Key Laboratory of Ministry of Education for Water Quality Security and Protection in Pearl River Delta, School of Environmental Science and Engineering, Guangzhou University, Guangzhou 510006, China; 7 Linköping University-Guangzhou University Research Center on Urban Sustainable Development, Guangzhou University, Guangzhou 510006, China; 8 Key Laboratory for City Cluster Environmental Safety and Green Development of the Ministry of Education, Institute of Environmental and Ecological Engineering, Guangdong University of Technology, Guangzhou 510006, China; fl_li@gdut.edu.cn; 9 Research Center for Eco-Environmental Sciences, Chinese Academy of Sciences, Beijing 100085, China; liuwb@rcees.ac.cn

**Keywords:** phthalate, dietary intake, DEHP, DBP, DIBP, bottled drink

## Abstract

Ubiquitous occurrences of phthalic acid esters (PAEs) or phthalates in a variety of consumer products have been demonstrated. Nevertheless, studies on their occurrence in various types of bottled drinks are limited. In this study, fifteen PAEs were analyzed in six categories of bottled drinks (*n* = 105) collected from the Chinese market, including mineral water, tea drinks, energy drinks, juice drinks, soft drinks, and beer. Among the 15 PAEs measured, DEHP was the most abundant phthalate with concentrations ranging from below the limit of quantification (LOQ) to 41,000 ng/L at a detection rate (DR) of 96%, followed by DIBP (DR: 88%) and DBP (DR: 84%) with respective concentration ranges of below LOQ to 16,000 and to 4900 ng/L. At least one PAE was detected in each drink sample, and the sum concentrations of 15 PAEs ranged from 770 to 48,004 ng/L (median: 6286 ng/L). Significant differences with respect to both PAE concentrations and composition profiles were observed between different types of bottled drinks. The median sum concentration of 15 PAEs in soft drinks was over five times higher than that detected in mineral water; different from other drink types. Besides DEHP, DBIP, and DBP, a high concentration of BMEP was also detected in a tea drink. The estimated daily dietary intake of phthalates (EDI_drink_) through the consumption of bottled drinks was calculated based on the concentrations measured and the daily ingestion rates of bottled drink items. The EDI_drink_ values for DMP, DEP, DIBP, DBP, BMEP, DAP, BEEP, BBP, DCP, DHP, BMPP, BBEP, DEHP, DOP, and DNP through the consumption of bottled mineral water (based on mean concentrations) were 0.45, 0.33, 12.5, 3.67, 2.10, 0.06, 0.32, 0.16, 0.10, 0.09, 0.05, 0.81, 112, 0.13, and 0.20 ng/kg-bw/d, respectively, for Chinese adults. Overall, the EDI_drink_ values calculated for phthalates through the consumption of bottled drinks were below the oral reference doses suggested by the United States Environmental Protection Agency (U.S. EPA).

## 1. Introduction

Phthalic acid esters (PAEs) or phthalates, primarily used to make polyvinyl chloride (PVC), or vinyl, flexible and pliant, are a group of chemicals made of aryl esters of phthalic acid and alkyl. PAEs are generally used to soften plastics because of their strong performance, durability, and stability [1,2]. These phthalate plasticizers are used in hundreds of products in our homes, hospitals, cars, and businesses, such as vinyl flooring, plastic packaging, toys, medical tubing, and cosmetics [3,4,5,6,7]. For example, Xu et al. (2020) reported the sum concentrations of dimethyl phthalate (DMP), diethyl phthalate (DEP), and dibutyl phthalate (DBP) ranged from 102 to 710 µg/kg in polyethylene terephthalate (PET) bottles collected from Beijing, China [8]. PAEs are not covalently bound to the plastic [9,10], so they can be easily released into the environment, leading to potential human exposure through ingestion, dermal absorption, and inhalation.

As a group of well-studied endocrine-disrupting chemicals, phthalates exposure has been associated with a variety of health effects, including premature thelarche, endometriosis, low semen quality, diabetes, overweight and obesity, allergy and asthma, and reproductive health [11,12]. Diethylhexyl phthalate (DEHP) is one of the most-studied phthalates, and accumulative evidence showed that DEHP exposure was significantly related to insulin resistance and higher systolic blood pressure as well as reproductive system problems [13,14]. Potential toxicity mechanisms of DEHP exposure include the activation of Kupffer’s cells and the nuclear receptor peroxisome proliferator-activated receptor (PPARα) [15,16,17]. Evidence has shown that phthalates’ toxicity heavily depends on their chemical structures [18]. Based on the difference in carbon backbones in the alkyl side chain, phthalates are differentiated into low and high molecular weight categories. Low molecular weight (LMW) phthalate plasticizers have straight carbon backbones of C3-C6 in the alkyl side chains, while high molecular weight (HMW) phthalate plasticizers have straight C7-C13 carbon backbones in the alkyl side chains [18]. Studies have indicated that LMW phthalates can cause adverse reproductive effects, while HMW phthalates and those C1-C2 backbone alkyl phthalates do not show adverse reproductive effects [18]. The US Environmental Protection Agency (EPA) listed DEHP and butyl benzyl phthalate (BBP) as probable and possible human carcinogens, respectively [11]. European authorities have also classified LMW phthalates with C3-C6 backbone alkyl phthalates as presumed human reproductive toxicants.

Parent phthalates and their metabolites have been detected in a variety of human samples, including serum [19], urine [20,21], semen [22], breast milk [23], and breast tumor tissue [24]. Considerable efforts have also been made to characterize the sources of human exposure to PAEs [25,26], and the ubiquitous occurrence of PAEs in both consumer products [25,26,27,28] and environmental matrices [29,30,31] has been reported. The accumulating evidence has shown that the sources and routes of human exposure to individual PAEs can vary depending on their physicochemical properties [26,31,32,33]. For example, cosmetics and personal care products are the major sources of human exposure to LMW phthalates [25], diet has been a major source of exposure to HMW phthalates, especially DEHP [31,32,34], and inhalation is the predominant exposure route to DMP [32]. Recent studies have indicated that drinking water is also an important source of human exposure to PAEs. For example, Liu et al. (2015) performed a national survey and risk assessment of phthalates in drinking water from waterworks in China and found that DBP and DEHP were the most abundant PAEs among the six PAEs measured at median levels of 0.18 ± 0.47 and 0.18 ± 0.97 µg/L, respectively [35]. Thuy et al. (2021) surveyed the contamination levels and distribution patterns of ten PAEs in various types of water samples, including bottled water and tap water, collected from Hanoi, Vietnam, and suggested widespread occurrence of PAEs in the water samples [36]. However, little is known of the occurrence of phthalates in bottled drinks commercially available in the market, although the consumption of bottled drinks is huge.

This study aims to investigate the occurrence and distribution levels of fifteen typical phthalates in 105 popular branded bottled drinks in the Chinese market, including mineral water (*n* = 19), a tea drink (*n* = 22), an energy drink (*n* = 15), a juice drink (*n* = 15), a soft drink (*n* = 25), and beer (*n* = 9), in order to estimate human exposure to phthalates through the consumption of bottled drinks. We also chemo-metrically investigate if grouping and correlations among PAEs and bottled drinks from the Chinese market exist. To our knowledge, this is the first survey on phthalates in various types of bottled drinks collected from the Chinese market.

## 2. Materials and Methods

### 2.1. Standards and Reagents

Fifteen phthalates, including dimethyl phthalate (DMP), diethyl phthalate (DEP), dibutyl phthalate (DBP), dinonyl phthalate (DNP), diamyl phthalate (DAP), dihexyl phthalate (DHP), diisobutyl phthalate (DIBP), butyl benzyl phthalate (BBP), bis(2-Ethoxyethyl) phthalate (BEEP), bis(2-methoxyethyl) phthalate (BMEP), bis(2-*n*-Butoxyethyl) phthalate (BBEP), bis(4-Methyl-2-pentyl)phthalate (BMPP), dinoctyl phthalate (DOP), dicyclohexyl phthalate (DCP), and diethylhexyl phthalate (DEHP), were analyzed in this study. Detailed information regarding the 15 PAEs is shown in Table S1. Nine deuterated internal standards, including d_4_-DMP, d_4_-DEP, d_4_-DBP, d_4_-DNP, d_4_-DHP, d_4_-DIBP, d_4_-DOP, d_4_-DCP, and d_4_-DEHP, were used as surrogate standards in the quantification of phthalates. Both the target and surrogate standards were purchased from AccuStandard, Inc. (New Haven, CT, USA), with a purity of >99%. Analytical-grade acetone and acetonitrile were purchased from Macron Chemicals (Nashville, TN, USA), and hexane and HPLC-grade water were purchased from J. T. Baker (Phillipsburg, NJ, USA).

### 2.2. Sample Collection and Analysis

A total of 105 bottled drinks were collected from local supermarkets in Dalian, Liaoning Province, China, including mineral water (*n* = 19), a tea drink (*n* = 22), an energy drink (*n* = 15), a juice drink (*n* = 15), a soft drink (*n* = 25), and beer (*n* = 9). The drink samples collected in this study were popular brands that were consumed widely by the Chinese population.

All the drink samples were spiked with surrogate standards prior to extraction, following the extraction protocol described earlier [28]. In brief, 200 ng of each surrogate standard was spiked into 1500 mL of the bottled drink sample. Spiked samples were thoroughly mixed for 5 min and allowed to equilibrate at room temperature for 30 min. Then 10 mL hexane was used for extraction via shaking in a mechanical shaker at 250 oscillations/min for 30 min. After centrifugation, the hexane layer was transferred into a clean glass flask. The extraction was repeated three times, and the hexane extract was combined and concentrated using a rotary evaporator to 1 mL and transferred into a gas chromatography (GC) vial for analysis.

The instrumental analysis protocol of phthalates was described elsewhere in earlier studies [28,31]. Briefly, the analysis was performed using GC (Agilent Technologies 6890, Santa Clara, CA, USA) coupled with mass spectrometry (Agilent Technologies 5973, Santa Clara, CA, USA) in the selection ion monitoring (SIM) mode. The chromatography separation was carried out using a fused silica capillary column (DB-5 ms, 30 m × 0.25 mm × 0.25 µm; Agilent Technologies; Santa Clara, CA, USA). The detailed parameters for the GC-MS condition for PAE analysis are shown in Table S2.

### 2.3. Quality Assurance/Quality Control

Prior to the analysis of samples, considerable effort was made to reduce the background contamination from the analytical procedures following the earlier studies [7]. Briefly, all glassware was washed with a detergent and Milli-Q water, followed by solvents (i.e., acetone and hexane), baked at 450 °C for overnight, and kept in an oven at 100 °C until use. All solvents were tested for background levels of phthalates and the batches of solvents that contained the lowest levels of phthalates were used throughout the analysis. Prior to each batch of analysis, pure hexane was injected into GC–MS until the background level was stable. Within each batch of ten samples, three solvent blanks and three procedural blanks and a pair of matrix-spike samples were processed together. Trace levels of phthalates found in procedural blanks were subtracted from the measured concentrations in bottled drink samples (Table S2). The quantification of phthalates in the samples was based on the isotope dilution method. The calibration curves were prepared by plotting a concentration−response factor for each target analyte (peak area of analyte divided by peak area of the internal standard) versus the response-dependent concentration factor (the concentration of the analyte divided by the concentration of the internal standard). The regression coefficients (r) were ≥ 0.99 for all calibration curves. The limits of quantification (LOQs) were calculated based on the instrument detection limits (a quantifiable peak must have a signal-to-noise ratio > 10, and a dilution factor in sample preparation (Table S3)). Recoveries of surrogate standards were calculated using matrix spikes of both low (50 ng each PAE) and high (500 ng each PAE) amounts of chemical spikes, and the recoveries ranged from 82% to 113% (Table S3). The relative standard deviation (RSD) was calculated by analyzing a high amount of matrix spike replicates (*n* = 3) to evaluate the reproducibility and repeatability of the analysis, and the RSDs of PAEs are below 10% (Table S3).

### 2.4. Statistical Analysis

Basic descriptive statistical analysis was performed using Microsoft Excel (Microsoft Office 2013). For concentrations below the LOQs, a value of half the LOQ was used in the calculation [37,38]. As a major tool for simplifying the large initial datasets, principal component analysis (PCA) has been widely used in the investigation of possible sources of chemical pollutants in the environment [38,39]. Here, Euclidean distance-based constrained analysis of principal coordinates (CAP), a type of principal component analysis, was used to provide information regarding the sources of PAEs in the analyzed bottled drink samples, and to compare the PAEs concentrations among different groups [40]. PRIMER-e (version 7, PRIMER-E, Ivybridge, UK) with PERMANOVA+ add-on software (PRIMER-E Ltd., Ivybridge, UK) was used in the PCA analysis, and the statistical significance level was set at α < 0.05.

### 2.5. Exposure Doses and Health Risk Assessment of PAEs through Consumption of Bottled Drinks

Daily intake of phthalates through the consumption of bottled drinks by the Chinese population was estimated via the following equation [31]:

(1) $$\mathrm{EDI}{\mathrm{drink}}_{\;}=\frac{CQ}{bw}$$

where EDI_drink_ (ng/kg-bw/d) is the estimated daily intake from drink, *C* (ng/g) is the phthalate concentration in the drink, *Q* (g/day) is the average amount of daily intake of the drink, and *bw* (kg) is body weight. For Chinese adults, 60 kg and 2000 g/day were used as the *bw* and Q values, respectively.

Both carcinogenic and non-carcinogenic risks of the select PAEs through the consumption of bottled drinks were assessed following the methods described earlier [41]. The selection of PAEs was based on the availability of relevant parameters in the Integrated Risk Information System, prepared and maintained by the U.S. Environmental Protection Agency (U.S. EPA) [42]. The carcinogenic risk (R) from exposure to PAEs via the consumption of bottled drinks was calculated by the following equation:

(2) R=SF×EDI (R < 0.01)

(3) $$R=1-\exp\left(˗EDI\times SF\right)\;(R\;\geq \;0.01)$$

where *SF* is the carcinogenic slope factor of oral intake. The *SF* value for DEHP is 0.014 (Kg d)/mg [42].

The hazard index (*HI*) was used to assess non-cancer risks, calculated using the following equation:

(4) HI=EDI/RfD

where *RfD* is the reference dose for the non-carcinogenic health risk of a chemical proposed by the guidelines. The *RfD* of BBP, DBP, DEP, and DEHP is 0.2, 0.1, 0.8, and 0.02 mg/Kg/d, respectively [42]. HI below 1 indicates safety concerns [41].

## 3. Results and Discussion

### 3.1. Concentrations of Phthalates in Bottled Drinks

Overall, at least one PAE was detected in each one of the 105 bottled drinks analyzed (Table 1). The sum concentrations of 15 PAEs measured in the 105 bottled drink samples ranged from 770 to 48,004 ng/L at a median level of 6286 ng/L (Table 1). Among the 15 PAEs measured in this study, DEHP was the predominant compound detected in the bottled drinks (detection rate, DR: 96%; median: 2000 ng/L; range: <LOQ-41000 ng/L), followed by DIBP (88%; 2100; <LOQ-16000) and DBP (84%; 820; <LOQ-4900) (Table 1). All other PAEs were also frequently detected in this study with DRs over 59%, but their contributions to the sum mean concentration of the 15 PAEs were low, with a contribution ratio between 0.1% and 4.4% (Table 1).

Mineral water is the most commonly used bottled drink among the Chinese population. Among the 15 PAEs measured, DEHP was the most frequently detected and most-abundant chemical in the mineral water samples, with a DR of 100% and median concentration of 1600 (range: 500–15,000) ng/L, followed by DIBP (DR: 58%; median: 170; range: <LOQ-940) and DBP (37%; 57.0; <LOQ-320) (Table 2 and Table 3). The distribution pattern is similar to that observed when taking other types of bottled drinks into account (Table 1), but different from what was observed in bottled waters collected from other countries such as Vietnam [36]. Differences in packaging material, water source, and other materials used in the production and bottling between the two countries may explain this observation. When compared with the concentration in bottled water or tap water samples collected from other regions, the concentration of DEHP in mineral water was at a higher level (at the same level as that calculated when taking other types of drinks into account), but the concentrations of DIBP and DBP were at lower levels (DIBP and DBP levels were also at higher levels when taking other types of bottled drinks into account), as shown in Table 2. This indicates that bottled mineral water/drinks are an important source of human exposure to DEHP, similar to other dietary sources such as foodstuffs [28]. The sum concentrations of 15 PAEs measured in 19 bottled mineral water samples ranged from 770 to 16,301 ng/L (median: 1805 ng/L) (Table 3). Median individual PAE concentrations in the bottled drink samples (*n* = 105) were 1.3–16.1 times higher than that detected in the bottled mineral water samples (*n* = 19) (Table 1 and Table 3).

Concentrations of PAEs in both bottled mineral water samples and bottled drink samples analyzed in this study were at higher levels compared with those reported in other countries, e.g., Iran [43,45] and Portugal [46]. This may be partly ascribed to the fact that more types of phthalates (15 vs. 6 [43], 6 [45], and 11 [46], respectively) were measured in this study as well as the differences in the sample pretreatment method, analytical technique, data analysis method, etc. Le et al. (2021) [36] recently reported the concentrations of 10 typical PAEs in bottled water collected from Hanoi, Vietnam with the mean concentration being 6400 (range: 1640–15,700) ng/L, which is higher than that detected in mineral water yet lower than that in bottled drinks analyzed in this study. The PAE concentrations in the bottled drinks detected in this study are lower than that reported by Luo et al. (2018) [44] in bottled waters from 21 countries (mean: 14,900 ng/L; range: n.a.–520,000 ng/L).

Several studies also investigated the concentrations of PAEs in bottled water samples in China, but the reported concentrations are lower than those found in mineral water in this study. Liu et al. (2015) collected a total of 225 drinking water samples from the waterworks in different regions of China and determined the concentrations of six typical PAEs including DEP, DMP, DBP, BBP, DEHP, and DOP, and the mean sum concentration was 1278 ng/L [35] (mean concentration was 4015 ng/L for mineral water samples in this study). Wang et al. (2021) collected bottled water samples from Tianjin, China, and reported that the mean sum concentration of DBP, BBP, and DEHP was 1960 ng/L [41]. Li et al. (2019) reported the concentrations of seven PAEs (DMP, DEP, DPP, DBP, BzBP, DEHP, and DnOP) in 60 bottled water samples collected in Beijing, China, and the sum PAE concentrations ranged from 155 to 5200 (mean: 519) ng/L [47].

Overall, the concentration of individual PAE in bottled drinks did not exceed the maximum contaminant levels recommended by national and international authorities (e.g., in China, the guideline values for DEHP, DBP, and DEP are 8, 3, and 300 µg/L, respectively [48]; the guideline value for DEHP in WHO [49] and the U.S. [50] are 8 and 6 µg/L, respectively). However, the concentration of individual PAE in select bottled drinks may exceed the no-observed-adverse-effect level (NOAEL) suggested by U.S. EPA (e.g., NOAELs for BBP and DEHP in water are 0.10 and 0.32 µg/L, respectively [51]). We further calculated the hazard index (HI) for DEHP using the highest concentration of DEHP (41000 ng/L) observed in this study. The results showed that the highest HI of DEHP is 0.07, far less than 1, indicating that DEHP in bottled drinks posed negligible non-carcinogenic health risks to human health by ingestion. However, this still warrants attention when performing health risk assessment of chemical exposure because individuals are exposed to thousands of chemicals simultaneously and they may work synergistically in posing risks to human health.

### 3.2. Factors Influencing Phthalates Concentrations in Bottled Drinks

The bottled drinks analyzed in this study were grouped into six different types of bottled drinks, including (1) mineral water, (2) tea drink, (3) energy drink, (4) juice drink, (5) soft drink, and (6) beer. Compared with other types of drinks, mineral water samples contain the least phthalates with respect to both DRs and concentrations. Of the 15 PAEs measured in this study, only DEHP was detected in over 60% of mineral water samples (DR: 100%); however, in tea drink, energy drink, juice drink, soft drink, and beer samples, the number of PAEs with DRs over 60% was 14, 14, 15, 14, and 14, respectively (Table 3). With respect to concentrations of PAEs, of the six types of bottled drinks, soft drink had the highest sum concentration of 15 PAEs (range: 1991–48,004 ng/L; median: 10,534 ng/L), followed by juice drink (1635–46,541; 10,179), tea drink (1277–27,298; 8459), beer (1895–9104; 4111), energy drink (1184–36,505; 3972), and mineral water (770–16,301; 1805) (Table 3). The median sum concentration of 15 PAEs detected in soft drink samples is over five times higher than that detected in mineral water samples. Thus, considerable differences between the concentrations of PAEs in different types of bottled drinks were observed in this study. This is the first study showing that drink type can significantly impact the concentrations of PAEs in bottled drinks.

**Figure 1:**
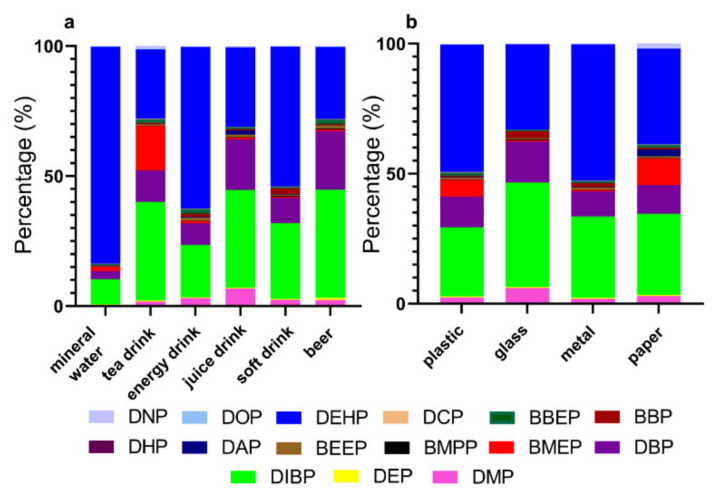
Compositions of total phthalates in different categories of bottled drinks: (**a**) Sorted by bottled drink types; (**b**) sorted by packaging material of bottled drinks.

To investigate the contribution of each phthalate to the total phthalate burden, we calculated the ratio of the mean concentration of each phthalate to the mean sum concentration of 15 PAEs (Table 3; Figure 1a). As shown in Table 3, DEHP, DBIP, and DBP are the three major PAEs detected in beer, soft drink, juice drink, and energy drink samples, with a contribution ratio of over 10% (or around 10%). The predominant compounds found in the four types of bottled drinks are DIBP, DEHP, DIBP, and DEHP, respectively, with the respective contribution ratios being 41.7%, 53.8%, 37.5%, and 62.1%. In mineral water samples, DEHP is the predominant PAE with a contribution ratio of 84%, followed by DBIP (ratio: 9.4%), and the contribution of DBP is minor (2.8%). In tea drink samples, besides DEHP, DBIP, and DBP, we also observed a significant contribution of BMEP to the sum PAE concentration with a contribution ratio of 17.1% (Table 2 and Table 3). This indicates that tea drink is an important source of human exposure to BMEP.

We further examined the concentrations of PAEs in bottled drinks based on the packaging material, including plastic (*n* = 56), glass (*n* = 19), metal (*n* = 22), and paper (*n* = 8) (Table 4; Figure 1b). As shown in Table 4, of the 15 PAEs measured, the majority of chemicals (12–14) had DRs over 60% in each category. DEHP, DBIP, and DBP are the predominant phthalates found in each category with corresponding contribution ratios of over 10%. Compared with other packaging materials, paper-bottled drinks have a higher concentration of BMEP with a contribution ratio of 10.2%. The highest sum concentration of the 15 PAEs was found in paper-bottled drinks (range: 6418–46,541 ng/L; median: 11119 ng/L), followed by glass-bottled drinks (1635–23,256; 10,190), metal-bottled drinks (3078–48,004; 7501), and plastic-bottled drinks (770–27,298; 4340). This is different from our assumption that plastic may contain higher amounts of PAEs, which might be explained by the following reasons. Firstly, the sample size not large enough to investigate the impact of packaging material on PAEs concentrations within the same drink type. Secondly, even within the same type of packaging material, various sub-types exist. For example, different vendors may use different types of plastic in bottling the drinks. PAE concentrations may vary significantly depending on the specific plastic employed.

### 3.3. Principal Component Analysis (PCA) of Phthalates in Bottled Drinks

PCA was applied to provide information regarding the possible sources of PAEs detected in the bottled drink samples in Dalian, China. Here, we performed a canonical analysis of the principal coordinates (CAP) method to analyze the input dataset after log-transformation and standardization. CAP allows a constrained ordination to be done on the basis of any distance or dissimilarity measure. The analytical results on PAEs present in 105 bottled drink samples showed that the top six principal components, abbreviated as CAP here, explained 78.3% of the total variance in the data, with the top two CAPs explaining 37.3% and 11.0% variance, respectively. The percentages of the total variance explained by other CAPs are all below 10%. This indicates that there is only one major source of PAEs present in the bottled drinks, and a variety of factors are contributing to the PAEs concentrations in the bottled drinks analyzed in this study.

The correlation coefficients between the new abstract principal components and the PAEs were also provided, indicating how well the new abstract principal components correlate with the PAEs (Table S4). The first new abstract principal component, CAP1, correlates positively with all the PAEs measured in this study, implying that higher concentrations of PAEs were linked to higher values of CAP1. This could be explained by the same exposure sources of PAEs present in these bottled drinks.

**Figure 2:**
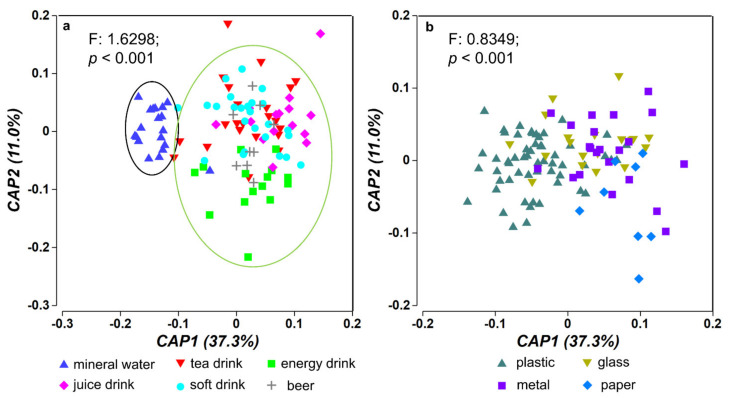
Plots of the canonical analysis of principal (CAP) of PAEs among different types of bottled drinks (**a**) and bottled drinks with different packaging materials (**b**). Permutational multivariate analysis of variance results are also shown in the figure.

Permutational multivariate analysis of variance was carried out to compare PAEs concentrations among different types of drinks, and a significant difference (*p* < 0.001) was observed, especially between mineral water and other types of drinks, as shown in Figure 2a. In addition, results of permutational multivariate analysis of variance also indicated the significant difference (*p* < 0.001) of the PAE concentrations among bottled drinks with different packaging materials (Figure 2b). Thus, both drink type and packaging material are associated with the PAEs in the samples. This further corroborated the earlier conclusion that many factors contribute to the PAEs present in the bottled drinks.

### 3.4. Dietary Exposure to PAEs through Consumption of Bottled Drinks in China

The human exposure doses of 15 PAEs through the ingestion of bottled drinks were estimated based on the mean/maximum concentrations of PAEs measured in different types of bottled drinks, as shown in Table 5. The average daily intake of drink for Chinese adults was estimated as 1 L per day [41]. Mineral water is the most commonly used bottled drink among the Chinese population. Among PAEs, the mean exposure doses of DEHP were the highest from the consumption of mineral water (mean/maximum dose: 112/500 ng/kg-bw/d), followed by DIBP (12.5/31.3) and DBP (3.67/10.7). The mean/maximum human exposure doses from mineral water for other PAEs (DMP, DEP, BMEP, DAP, BEEP, BBP, DCP, DHP, BMPP, BBEP, DOP, and DNP) were 0.45/1.03, 0.33/1.40, 2.10/10.3, 0.06/0.20, 0.32/3.17, 0.16/1.33, 0.10/0.19, 0.09/0.63, 0.05/0.08, 0.81/7.67, 0.13/0.40, and 0.20/0.63 ng/kg-bw/d, respectively. The mean/maximum human exposure doses to the total phthalates were 133/544 ng/kg-bw/d.

Of the six types of bottled drinks analyzed, the highest mean exposure doses of DEHP (240/1367), DIBP (151/533), and DBP (78.8/163) can be obtained through the consumption of soft drinks, juice drinks, and juice drinks, respectively. Other high exposure doses of individual PAEs include DMP though juice drinks (27.3/243) and BMEP through tea drinks (56.7/567). Based on the highest mean exposure doses of each PAE, it can be generalized that the EDI_drink_ values were in the order of 0.10 ng/kg-bw/d for DCP, DHP, BMPP, and DOP, 1.00 ng/kg-bw/d for DAP, BBP, DNP, DMP, DEP, BEEP, and BBEP, 10.0 ng/kg-bw/d for BMEP and DBP, and 100 ng/kg-bw/d for DIBP and DEHP.

Human exposure doses of PAEs through the consumption of bottled drinks were several orders of magnitude lower than the oral reference doses suggested by the U.S. FDA (20, 100, 200, and 800 µg/kg-bw/d for DEHP, DBP, BBP, and DEP, respectively) [52], even when the highest phthalate concentrations in bottled drinks were used in the estimation. However, humans are exposed to PAEs via multiple pathways including inhalation, diet ingestion, and dermal absorption. The evidence has shown that dietary exposures represent a small fraction of the total exposure doses (e.g., contributed ~10% for DBP, ~10% for DMP, and ~2% for DEP to the total exposures) [28]. Other exposure sources such as personal care products also play crucial roles in human exposure to phthalates. Thus, it is highly likely that the entire human exposure doses to phthalates for individuals might exceed the oral reference doses recommended by the U.S. FDA.

### 3.5. Health Risk Assessment of Select PAEs through Consumption of Bottled Drinks in China

Human cancer risk caused by DEHP via consumption of different types of bottled drinks was assessed by calculating the carcinogenic risk (R). Based on the mean concentrations of DEHP detected in different types of bottled drinks, the cancer risks of DEHP for mineral water, tea drink, energy drink, juice drink, soft drink, and beer are 1.6 × 10^−6^, 1.2 × 10^−6^, 2.2 × 10^−6^, 1.7 × 10^−6^, 3.4 × 10^−6^, and 0.6 × 10^−6^, respectively. Except for beer, the cancer risks of DEHP for other types of bottled drinks are higher than the maximum acceptable risk level, which is 1.0 × 10^−6^ [38]. Thus, the potential carcinogenic risk attributable to DEHP present in the bottled drink samples should be of concern for Chinese consumers. Consumption of bottled drinks over a long duration could be harmful to human health.

Non-carcinogenic risks of DEHP, DBP, DEP, and BBP were also evaluated via the calculation of HIs. The results showed that mean HIs for DEHP, DBP, DEP, and BBP were 5.6 × 10^−3^, 3.7 × 10^−5^, 0.4 × 10^−6^, and 0.8 × 10^−6^, respectively. These values are far less than 1, indicating that these PAEs in the bottled drinks collected in this study posed negligible non-carcinogenic health risks to human health by ingestion [38]. DEHP is the major chemical contributing to the non-carcinogenic risk of PAEs on average, posing non-carcinogenic risk two orders of magnitude higher than that of DBP. Because non-carcinogenic risk is highly associated with the concentrations of PAEs detected in the samples [38], the risk posed by other non-assessed chemicals (e.g., DIBP) is most likely much lower than DEHP.

It has been known that storage time and temperature can significantly impact the migration of chemicals from packaging material to drinks [38]. When bottled drinks are stored at a high temperature for a long time, human health risks posed by the ingestion of chemicals can be increased significantly, especially the carcinogenic risk [38]. Further, co-exposure of a variety of chemical pollutants under long-term chronic exposure may have a considerable total risk to human health. Therefore, the consumption of bottled drinks could be a non-neglectable risk factor contributing to human health risk.

## 4. Conclusions

In summary, this is the first study to investigate the occurrence and distribution of fifteen PAEs in various types of bottled drinks in China. Our results indicated the widespread occurrence of PAEs in different types of bottled drinks. Drink type is an important factor determining the concentrations of PAEs in the drinks. Significant differences of PAE concentrations between different types of bottled drinks were observed in this study. For example, the median sum concentration of 15 PAEs in soft drink samples is over five times higher than that detected in mineral water samples. Although human exposure doses of PAEs through the consumption of bottled drinks are much lower than the oral reference doses recommended by U.S. EPA, it is non-neglectable, especially considering the high frequency of the consumption of bottled drinks in daily life. Further, the higher carcinogenic risk posed by DEHP exposure through the consumption of bottled drinks warrants attention from the public.

Our results provide baseline information, for the first time, regarding the occurrence of PAEs in bottled drinks available in the Chinese market, which is helpful for people in choosing appropriate bottled drinks. To minimize PAE exposure, it is recommended to use bottled mineral water, instead of energy drinks, juice drinks, soft drinks, tea drinks, and beer, and avoid the use of bottled drinks with long-term storage at a high temperature. Compared with bottled drinks, tap water is recommended in everyday life. This is especially important for vulnerable members in the community, such as pregnant women, lactating women, infants, and children. Further, it is recommended to develop safer alternatives for DEHP, which is the most frequently observed PAE and can pose a higher carcinogenic risk. Authorities need to take measures to control the content of DEHP present in bottled drinks.

## Figures and Tables

**Table 1:** Overall concentrations of phthalates in bottled drinks (*n* = 105) collected from Dalian, Liaoning Province, China.

Chemical	DR^a^ (%)	Mean (ng/L)	SD^b^ (ng/L)	GM^c^ (ng/L)	Median (ng/L)	Range (ng/L)	Ratio^d^ (%)
DMP	75.2	277	837	64.9	65	<LOQ^e^–7300	3.0
DEP	59.0	32.9	44.2	20.3	17	<LOQ–390	0.4
DIBP	87.6	2825	2796	1491	2100	<LOQ–16000	30.7
DBP	83.8	1097	1119	551	820	<LOQ–4900	11.9
BMEP	87.6	404	2074	36.5	39	<LOQ–17000	4.4
DAP	61.0	41.0	174	5.12	3.5	<LOQ–1400	0.5
BEEP	84.8	63.4	61.2	32.8	50	<LOQ–270	0.7
BBP	81.9	97.6	464	13.3	12	<LOQ–3800	1.1
DCP	76.2	14.6	15.8	9.4	9.5	<LOQ–110	0.2
DHP	60.0	6.88	8.46	4.16	3.7	<LOQ–51	0.1
BMPP	75.2	16.4	39.2	6.6	6.2	<LOQ–280	0.2
BBEP	87.6	92.0	111	48.4	61	<LOQ–760	1.0
DEHP	96.2	4193	6949	2025	2000	<LOQ–41000	45.5
DOP	78.1	10.6	13.0	6.28	7	<LOQ–86.0	0.1
DNP	81.0	35.9	137	9.45	9.8	<LOQ–1300	0.4
∑(sum)	100	9207	8952	6127	6286	770–48004	

^a^: DR, detection rate; ^b^: SD, standard deviation; ^c^: GM, geometric mean; ^d^: ratio, concentration ratio (%), calculated as the ratio between the mean concentration of each target analyte versus the mean sum concentration of 15 PAEs; ^e^: LOQ, limit of quantification.

**Table 2:** Concentration^a^ (ng/L) comparison of DEHP, DIBP, DBP, and BMEP in bottled drink, bottled water, and tap water samples from various studies.

Sample Type	Location	*n*	DEHP	DIBP	DBP	BMEP	Reference
mineral water	Dalian, China	19	3351 (500–15000)	375 (<LOQ^b^-940)	110 (<LOQ-320)	63 (<LOQ-310)	this study
tea drink	Dalian, China	22	2660 (500–12000)	3770 (<LOQ-9900)	1197 (<LOQ-3600)	1701 (<LOQ-17000)	this study
energy drink	Dalian, China	15	4738 (<LOQ-34000)	1539 (<LOQ-4300)	630 (<LOQ-2900)	63 (<LOQ-220)	this study
juice drink	Dalian, China	15	3682 (440–27000)	4521 (240–16000)	2363 (290–4900)	73 (8.9–190)	this study
soft drink	Dalian, China	25	7198 (<LOQ-41000)	3916 (1000–7200)	1290 (93–3000)	56 (<LOQ-330)	this study
beer	Dalian, China	9	1317 (440–3700)	1974 (330–4100)	1064 (210–3000)	37 ((<LOQ-130)	this study
total	Dalian, China	105	4193 (<LOQ-41000)	2825 (<LOQ-16000)	1097 (<LOQ-4900)	404 (<LOQ-17000)	this study
non-carbonated water	Hanoi, Vietnam	11	873 (227–1950)	1100 (94.0–3930)	1150 (145–3070)	-	Le et al. (2021) [36]
carbonated water	Hanoi, Vietnam	10	1120 (103–2710)	1790 (123–5190)	1740 (93.0–4710)	-	Le et al. (2021) [36]
carbonated soft drinks	Tehran, Iran	4	8423 (6767–14008)	-	-	-	Moazzen et al. (2018) [43]
bottled water	Tianjin, China	6	1074 (880–1257)	-	486 (465–517)	-	Wang et al. (2021) [41]
bottled water	21 global countries	367–379	3420 (nd^c^-9410)	-	5350 (nd-2220)	-	Luo et al. (2018) [44]
bottled water	Tehran, Iran	10	100 (70–120)	-	70 (nd-120)	-	Abtahi et al. (2019) [45]
bottled water	Portugal	7	100 (20–180)	959 (100–1890)	1574 (60–6500)	-	Santana et al. (2013) [46]
tap water	Tehran, Iran	40	150 (nd-380)	-	90 (nd-140)	-	Abtahi et al. (2019) [45]
tap water	Hanoi, Vietnam	7	5340 (1010–14500)	456 (27.0–1390)	796 (14.0–2560)	-	Le et al. (2021) [36]
tap water	China	225	770 (<LOQ-5510)	-	350 (<LOQ-1560)	-	Liu et al. (2015) [35]
tap water	Tianjin, China	6	1338 (1097–1780)	-	541 (380–679)	-	Wang et al. (2021) [41]

^a^: mean concentration and the concentration range were used in this Table; ^b^: LOQ, limit of quantification; ^c^: nd, non-detected.

**Table 3:** Concentrations (ng/L) of phthalates in different types of bottled drinks.

	DMP	DEP	DIBP	DBP	BMEP	DAP	BEEP	BBP	DCP	DHP	BMPP	BBEP	DEHP	DOP	DNP	∑(sum)
mineral water (*n* = 19)																
DR^a^	26	5	58	37	53	5	32	42	21	11	5	53	100	47	53	100
mean	13.7	9.8	375	110	62.9	1.8	9.5	4.9	3.1	2.6	1.5	24.2	3351	3.8	5.9	3980
SD^b^	6.7	7.8	337	80.8	103	1.0	20.9	8.7	1.0	4.0	0.2	51.2	4321	3.0	5.4	4637
GM^c^	12.6	8.7	249	89.3	13.7	1.7	4.8	3.0	2.9	1.9	1.5	10.8	1989	3.0	3.8	2546
median	10.0	8.0	170	57.0	7.0	1.6	3.1	1.9	2.6	1.6	1.5	6.5	1600	1.6	4.4	1805
range	<LOQ^e^-31.0	<LOQ-42.0	<LOQ-940	<LOQ-320	<LOQ-310	<LOQ-6.00	<LOQ-95.0	<LOQ-40.0	<LOQ-5.80	<LOQ-19.0	<LOQ-2.30	<LOQ-230	500–15,000	<LOQ-12.0	<LOQ-19.0	770–16,301
ratio^d^	0.3	0.3	9.4	2.8	1.6	0.1	0.2	0.1	0.1	0.1	0.04	0.6	84.2	0.1	0.2	
tea drink (*n* = 22)																
DR	77	86	82	91	95	77	91	82	77	55	86	95	100	73	86	100
mean	177	38.1	3770	1197	1701	12.8	53.5	26.8	22.6	7.5	12.7	143	2660	16.0	105	9942
SD	291	30.0	2712	1037	4366	21.6	44.4	40.5	23.7	11.1	14.3	193	2380	21.4	274	7045
GM	68.2	28.4	2092	673	100	6.1	31.5	12.7	13.4	3.9	7.0	72.5	2043	7.2	21.6	7553
median	86.0	28.5	3550	1060	79.5	5.6	45.5	15.0	20.0	2.5	6.0	74.5	2050	7.4	25.0	8459
range	<LOQ-1300	<LOQ-110	<LOQ-9900	<LOQ-3600	<LOQ-17,000	<LOQ-100	<LOQ-150	<LOQ-190	<LOQ-110	<LOQ-49	<LOQ-55.0	<LOQ-760	500–12,000	<LOQ-86.0	<LOQ-1300	1277–27,298
ratio	1.8	0.4	37.9	12.0	17.1	0.1	0.5	0.3	0.2	0.1	0.1	1.4	26.8	0.2	1.1	
energy drink (*n* = 15)																
DR	87	53	93	80	93	73	100	100	100	100	93	100	80	100	67	100
mean	232	27.5	1539	630	63.3	13.7	94.7	80.8	19.1	10.5	13.7	140	4738	17.4	10.1	7629
SD	304	26.6	955	700	64.5	37.8	62.1	214	10.0	5.6	10.3	56.2	9893	14.2	13.6	10,072
GM	104	18.4	1230	372	37.9	4.4	78.4	26.2	16.9	9.1	10.0	129	1276	14.1	5.0	4688
median	98.0	14.0	1500	510	42.0	3.5	92.0	23.0	17.0	9.2	12.0	130	1100	14.0	3.0	3972
range	<LOQ-1100	<LOQ-98.0	<LOQ-4300	<LOQ-2900	<LOQ-220	<LOQ-150	22.0–270	5.7–850	9.1–37.0	3.7–20	<LOQ-37.0	52–250	<LOQ-34,000	6.2–63.0	<LOQ-53.0	1184–36,505
ratio	3.0	0.4	20.2	8.3	0.8	0.2	1.2	1.1	0.3	0.1	0.2	1.8	62.1	0.2	0.1	
juice drink (*n* = 15)																
DR	100	73	100	100	100	80	100	87	87	93	100	100	100	93	93	100
mean	820	38.2	4521	2363	72.9	202	115	51.5	16.2	9.2	16.9	88.6	3682	10.3	48.9	12,057
SD	1875	31.5	4654	1499	57.4	425	76.6	102	14.9	6.6	9.2	64.6	6617	8.2	125	11,012
GM	167	25.8	2631	1827	51.3	18.1	90.6	18.0	11.5	7.1	14.3	69.0	1823	7.7	16.5	8792
median	167	27	3400	2200	57	11	71	17	12	8.3	15	61	1600	8.7	14	10,179
range	18–7300	<LOQ-87	240–16,000	290–4900	8.9–190	<LOQ-1400	26.0–240	<LOQ-410	<LOQ-62	<LOQ-24	2.7–37.0	18–220	440–27,000	<LOQ-32	<LOQ-500	1635–46,541
ratio	6.8	0.3	37.5	19.6	0.6	1.7	1.0	0.4	0.1	0.1	0.1	0.7	30.5	0.1	0.4	
soft drink (*n* = 25)																
DR	84	68	100	100	96	72	96	96	92	56	84	88	96	84	96	100
mean	326	44.7	3916	1290	55.7	28.0	64.2	298	15.1	6.8	35.1	74.4	7200	8.6	15.6	13,376
SD	768	76.1	1957	826	71.8	70.9	58.9	917	14.2	10.7	76.0	59.7	9572	7.3	12.4	10,077
GM	92	24.3	3385	991	30.3	5.7	39.3	26.7	11.2	3.8	8.9	46.4	3705	6.0	11.2	10,710
median	110	22	3300	1100	36.0	4.6	39	20	10	3.4	7.0	66	2900	6.2	12.0	10,534
range	<LOQ-3100	<LOQ-390	1000–7200	93–3000	<LOQ-330	<LOQ-310	<LOQ-220	<LOQ-3800	<LOQ-71.0	<LOQ-51.0	<LOQ-280	<LOQ-210	<LOQ-41,000	<LOQ-30.0	<LOQ-52.0	1991–48,004
ratio	2.4	0.3	29.3	9.6	0.4	0.2	0.5	2.2	0.1	0.1	0.3	0.6	53.8	0.1	0.1	
beer (*n* = 9)																
DR	89	67	100	100	89	56	100	89	89	67	100	100	100	78	89	100
mean	113	35.8	1974	1064	36.8	5.3	61.0	13.1	8.2	4.7	8.3	84.0	1317	6.0	8.4	4740
SD	105	26.4	1157	861	38.6	4.4	32.8	10.8	4.3	3.1	6.3	97.8	1172	4.9	7.0	2374
GM	66.7	25.5	1572	801	21.9	3.9	54.2	9.7	7.2	3.7	7.0	52.0	989	4.5	6.0	4253
median	64	31	2000	980	30	5.0	52	8.3	7.1	4.9	6.8	59	780	4.9	6.7	4111
range	<LOQ-290	<LOQ-73	330–4100	210–3000	<LOQ-130	<LOQ-14.0	28.0–130	<LOQ-36.0	<LOQ-16.0	<LOQ-9.80	3.0–24.0	7.10–330	440–3700	<LOQ-17.0	<LOQ-20.0	1895–9104
ratio	2.4	0.8	41.7	22.5	0.8	0.1	1.3	0.3	0.2	0.1	0.2	1.8	27.8	0.1	0.2	

^a^: DR, detection rate (%); ^b^: SD, standard deviation (ng/L); ^c^: GM, geometric mean (ng/L); ^d^: ratio, concentration ratio (%), calculated as the ratio between the mean concentration of each target analyte versus the mean sum concentration of 15 PAEs; ^e^: LOQ, limit of quantification.

**Table 4:** Concentrations (ng/L) of phthalates in bottled drinks sorted by the packaging material.

	DMP	DEP	DIBP	DBP	BMEP	DAP	BEEP	BBP	DCP	DHP	BMPP	BBEP	DEHP	DOP	DNP	∑(sum)
plastic (*n* = 56)																
DR^a^	73	57	77	70	82	59	75	75	71	59	64	84	93	71	77	100
mean	217	33.7	1968	920	708	14.9	54.3	36.3	17.0	7.7	13.1	112	2826	12.8	24.9	6964
SD^b^	553	54.9	2205	1240	2816	45.3	51.6	115	18.4	8.8	23.2	136	3689	16.5	42.0	6421
GM^c^	53.7	19.6	861	336	44.1	4.6	25.2	10.5	10.2	4.5	5.7	54.0	1637	6.6	10.0	4559
median	45.0	17.0	1100	280	49.5	3.3	51.5	9.8	11.0	5.2	5.5	76.5	1650	7.4	11.0	4340
range	<LOQ^e^-3100	<LOQ-390	<LOQ-8100	<LOQ-4900	<LOQ-17,000	<LOQ- 310	<LOQ-220	<LOQ- 850	<LOQ-110	<LOQ-49.0	<LOQ-160	<LOQ-760	<LOQ-17,000	<LOQ-86.0	<LOQ-200	770–27,298
rat io^d^	3.1	0.5	28.3	13.2	10.2	0.2	0.8	0.5	0.2	0.1	0.2	1.6	40.6	0.2	0.4	
glass (*n* = 19)																
DR	79	53	100	100	95	63	90	84	79	68	79	90	100	74	79	100
mean	524	28.8	3510	1285	63.0	5.6	56.3	222	8.4	7.2	20.7	42.3	6083	5.4	10.2	11,871
SD	1653	26.4	3740	1042	75.9	5.6	75.7	867	5.7	11.2	55.9	31.5	9060	3.8	9.0	9584
GM	94.3	18.8	2085	853	33.1	3.9	29.4	15.9	6.8	4.2	6.7	28.9	2915	4.2	6.7	8418
median	109	12.0	2600	840	43.0	3.4	28.0	10.0	6.2	3.7	6.2	32.0	2600	4.1	8.4	10,190
range	<LOQ-7300	<LOQ- 80	240–16,000	<LOQ-3100	<LOQ-310	<LOQ-24.0	<LOQ-270	<LOQ-3800	<LOQ-22.0	<LOQ-51.0	<LOQ-250	<LOQ-100	440–34,000	<LOQ-13.0	<LOQ-36.0	1635–36,505
ratio	4.4	0.2	29.6	10.8	0.5	0.1	0.5	1.9	0.1	0.1	0.2	0.4	51.2	0.1	0.1	
metal (*n* = 22)																
DR	82	55	100	100	91	59	100	91	86	55	91	91	100	91	91	
mean	248	29.9	3216	1136	42.9	20.9	80.6	155	13.8	4.9	22.7	89.5	5502	10.1	12.3	10,584
SD	486	29.1	1469	804	72.3	45.8	56.8	595	15.0	4.6	58.2	84.7	9848	8.2	11.1	10,841
GM	72.0	19.4	2858	919	21.7	4.94	589	15.8	9.63	3.4	8.6	53.7	2220	7.3	7.6	7782
median	58.0	17.0	3200	970	25.5	3.65	63.0	12.5	9.35	2.8	7.9	59.0	1400	6.3	8.4	7501
range	<LOQ-2100	<LOQ-98.0	750- 5800	240–3300	<LOQ-330	<LOQ- 170	4.2–220	<LOQ-2800	<LOQ-71.0	<LOQ-16.0	<LOQ-280	<LOQ-330	470–41,000	<LOQ-30.0	<LOQ-36.0	3078–48,004
ratio	2.3	0.3	30.4	10.7	0.4	0.2	0.8	1.5	0.1	0.1	0.2	0.9	52.0	0.1	0.1	
paper (*n* = 8)																
DR	75	100	100	100	100	63	100	100	75	63	100	100	100	100	88	100
mean	197	44.5	6125	1780	74.5	363	96.4	73.5	14.9	6.0	11.5	78.1	5680	8.7	238	14,791
SD	245	28.8	3925	962	60.8	546	85.3	138	11.2	7.6	9.9	63.6	8714	4.0	462	13,292
GM	76.9	35.2	5252	1531	51.0	23.5	54.7	27.3	9.9	3.8	8.2	57.2	2932	7.8	25.8	11,815
median	109	44.5	4900	1750	66.0	5.6	82.5	21	16	3.2	6.0	57.0	3550	8.2	8.5	11,119
range	<LOQ-700	14- 80	2900–14,000	480- 3600	11- 190	<LOQ-1400	4.80–240	5.90- 410	<LOQ-28.0	<LOQ-24.0	2.70–28.0	15.0–180	440–27,000	2.7–14.0	<LOQ -1300	6418–46,541
ratio	1.3	0.3	41.4	12.0	0.5	2.5	0.7	0.5	0.1	0.04	0.1	0.5	38.4	0.1	1.6	

^a^: DR, detection rate (%); ^b^: SD, standard deviation (ng/L); ^c^: GM, geometric mean (ng/L); ^d^: ratio, concentration ratio (%), calculated as the ratio between the mean concentration of each target analyte versus the mean sum concentration of 15 PAEs; ^e^: LOQ, limit of quantification.

**Table 5:** Estimated daily intake (EDI_drink_, ng/kg-bw/d) of PAEs through ingestion of bottled drinks, based on mean/maximum concentrations.

Chemical	Mineral Water	Energy Drink	Beer	Tea Drink	Juice Drink	Soft Drink
DMP	0.45/1.03	7.72/36.7	3.77/9.67	5.91/43.3	27.3/243	10.9/103
DEP	0.33/1.40	0.92/3.27	1.19/2.43	1.27/3.67	1.27/2.90	1.49/13.0
DIBP	12.5/31.3	51.3/143	65.8/137	126/330	151/533	131/240
DBP	3.67/10.7	21.0/96.7	35.5/100	39.9/120	78.8/163	43.0/100
BMEP	2.10/10.3	2.11/7.33	1.23/4.33	56.7/567	2.43/6.33	1.860/11.0
DAP	0.06/0.20	0.46/5.00	0.18/0.47	0.42/3.33	6.75/46.7	0.93/10.3
BEEP	0.32/3.17	3.16/9.00	2.03/4.33	1.78/5.00	3.82/8.00	2.14/7.33
BBP	0.16/1.33	2.69/28.3	0.44/1.20	0.89/6.33	1.72/13.7	9.95/127
DCP	0.10/0.19	0.64/1.23	0.27/0.53	0.75/3.67	0.54/2.07	0.50/2.37
DHP	0.09/0.63	0.35/0.67	0.16/0.33	0.25/1.63	0.31/0.80	0.23/1.70
BMPP	0.05/0.08	0.46/1.23	0.28/0.80	0.42/1.83	0.56/1.23	1.17/9.33
BBEP	0.81/7.67	4.66/8.33	2.80/11.0	4.78/25.3	2.95/7.33	2.48/7.00
DEHP	112/500	158/1133	43.9/123	88.7/400	123/900	240/1367
DOP	0.13/0.40	0.58/2.10	0.20/0.57	0.53/2.87	0.34/1.07	0.29/1.00
DNP	0.20/0.63	0.34/1.77	0.28/0.67	3.49/43.3	1.63/16.7	0.52/1.73
∑(sum)	133/544	254/1217	158/304	331/910	402/1551	446/1600

## Data Availability

Data is contained within the article or supplementary material.

## Supplementary Materials

The following are available online, Table S1: title, Detailed information of the 15 PAEs measured in this study; Table S2: title, Instrumental parameters on GC-MS conditions for phthalate analysis; Table S3: title, Concentrations (ng/L) of the target phthalates in procedural blanks and their limits of detection (LOD) and limits of quantification (LOQ); Table S4: title, Correlation coefficients between the new abstract principal components and the PAEs present in the bottled drinks.

## Sample Availability

Samples analyzed in this study can be purchased from the Chinese market.
