# Supplementary material for: Occurrence of Phthalates in Bottled Drinks in the Chinese Market and Its Implications for Dietary Exposure
Source: Molecules. 2021 Oct 6;26(19):6054. doi: 10.3390/molecules26196054 (PMC8511988; doi:10.3390/molecules26196054)
Supplement: Supplementary file 1 [file molecules-26-06054-s001.zip › molecules-1401040-supplementary.pdf]

Occurrence of Phthalates in bottled drinks in Chinese market and its implications for dietary exposure

Xiaohong Xue[1], Yaoming Su[2], Hailei Su[3], Dongping Fan[2], Hongliang Jia[4], Xiaoting Chu[4], Xiaoyang Song[5], Yuxian Liu[6,7*], Feilong Li[8], Jingchuan Xue[8]*, Wenbin Liu[9]

[1]College of Science, Dalian Maritime University, 1 Linghai Road, Dalian, 116026 Liaoning, China
[2]South China Institute of Environmental Sciences, Ministry of Ecology and Environment of P.R.C., No. 7, West Street, Yuancun, Tianhe District, Guangzhou, 510535, China
[3]State Key Laboratory of Environmental Criteria and Risk Assessment, Chinese Research Academy of Environmental Sciences, Beijing, 100012, China
[4]International Joint Research Centre for Persistent Toxic Substances (IJRC-PTS), College of Environmental Science and Engineering, Dalian Maritime University, Dalian 116026, China
[5]Dalian Modern Agricultural Production Development Service Center, 678 Zhongshan Road, Dalian, 116026 Liaoning, China
[6]Key Laboratory of Ministry of Education for Water Quality Security and Protection in Pearl River Delta, School of Environmental Science and Engineering, Guangzhou University, Guangzhou 510006, China
[7]Linköping University-Guangzhou University Research Center on Urban Sustainable Development, Guangzhou University, Guangzhou 510006, China
[8]Key Laboratory for City Cluster Environmental Safety and Green Development of the Ministry of Education, Institute of Environmental and Ecological Engineering, Guangdong University of Technology, Guangzhou, 510006, China
[9]Research Center for Eco-Environmental Sciences, Chinese Academy of Sciences, Beijing, 100085, China

*Correspondence author
Yuxian Liu: School of Environmental Science and Engineering, Guangzhou University, email: liuyuxian@gzhu.edu.cn
Jingchuan Xue: Institute of Environmental and Ecological Engineering, Guangdong University of Technology, email: xue@gdut.edu.cn

Table S1. Detailed information of the 15 PAEs measured in this study.

full name	abbreviation	chemical formula	molecular weight	Log Kow
low molecular weight phthalates				
dimethyl phthalate	DMP	$C_{10}H_{10}O_4$	194.2	1.6
diethyl phthalate	DEP	$C_{12}H_{14}O_4$	222.2	2.47
dibutyl phthalate	DBP	$C_{16}H_{22}O_4$	278.4	4.5
diisobutyl phthalate	DIBP	$C_{16}H_{22}O_4$	278.4	4.11
bis(2-methoxyethyl) phthalate	BMEP	$C_{14}H_{18}O_6$	282.3	1.11
diamyl phthalate	DAP	$C_{18}H_{26}O_4$	306.4	5.62
bis(2-ethoxyethyl) phthalate	BEEP	$C_{16}H_{22}O_6$	310.3	2.1
butyl benzyl phthalate	BBP	$C_{19}H_{20}O_4$	312.4	4.73
dicyclohexyl phthalate	DCP	$C_{20}H_{26}O_4$	330.4	6.2
dihexyl phthalate	DHP	$C_{20}H_{30}O_4$	334.4	6.82
bis(4-methyl-2-pentyl) phthalate	BMPP	$C_{20}H_{30}O_4$	334.4	
bis(2-n-butoxyethyl) phthalate	BBEP	$C_{20}H_{30}O_6$	366.4	4.06
diethylhexyl phthalate	DEHP	$C_{24}H_{38}O_4$	390.6	7.6
high molecular weight phthalates				
dinoctyl phthalate	DOP	$C_{24}H_{38}O_4$	390.6	8.1
dinonyl phthalate	DNP	$C_{26}H_{42}O_4$	418.6	9.52

Table S2. Instrumental parameters on GC-MS conditions for phthalate analysis

Parameter	***Condition/setting values***		
Instrument	GC 6890B MS 5973 (Agilent Technologies)		
Column	DB-5MS (30 m × 0.25 mm × 0.25 μm; Agilent Technologies)		
Injection	Splitless mode, injection volume 2 μL, injection port temperature 280°C		
Carrier gas	Helium, flow rate 0.5 mL/min		
Oven temperature program	80 ^{o}C (hold 1 min), to 180 ^{o}C (12 ^{o}C/min, hold 1 min), to 230 ^{o}C (6 ^{o}C/min), to 270 ^{o}C (8 ^{o}C/min, hold 2 min), to 300 ^{o}C (20 ^{o}C/min, hold 15 min)		
Interface temperature	250 ^{o}C	Ion source temperature	230 ^{o}C
Ionization mode	EI, electron energy 70 eV	Monitoring mode	SIM

Table S3. Concentrations (ng/L) of the target phthalates in procedural blanks and their limits of detection (LOD) and limits of quantification (LOQ).

chemical	BLK[a]	LOQ[b]	relative recovery (%)		RSD(%)[e]
			low[c]	high[d]	
DMP	<LOQ	21	94.6±7.14	94.6±7.63	8.43
DEP	<LOQ	16	95.8±4.73	93.8±6.02	4.26
DIBP	<LOQ	214	104±8.03	104±6.03	5.06
DBP	<LOQ	113	107±8.47	103±4.80	5.37
BMEP	<LOQ	6	90.6±4.58	96.0±4.04	4.72
BMPP	<LOQ	3	93.4±5.39	94.6±5.04	4.08
BEEP	<LOQ	6	89.3±6.82	92.6±5.84	6.32
DAP	<LOQ	3	89.5±8.53	95.8±3.48	2.48
DHP	<LOQ	3	86.4±1.58	87.4±4.20	5.39
BBP	<LOQ	4	84.6±9.04	89.1±5.47	9.52
BBEP	<LOQ	9	90.6±11.2	95.2±8.65	9.58
DCP	<LOQ	5	94.8±10.3	93.8±6.32	6.04
DEHP	258±24	212	110.4±8.57	106±7.53	7.28
DOP	<LOQ	3	106±6.54	104±5.06	5.43
DNP	<LOQ	3	97.9±2.45	96.3±4.06	4.68

[a]: BLK, procedural blank, calculated by analyzing the PAEs in the procedural blank samples (n=3); [b]: LOQ, limit of quantification; [c]: relative recoveries of PAEs in the analysis of bottled drink samples calculated using matrix spike of low amount chemicals (50 ng); [d]: relative recoveries of PAEs in the analysis of bottled drink samples calculated using matrix spike of high amount chemicals (500 ng); [e]: RSD, relative standard deviation, calculated using the replicates analysis of high amount chemical matrix spikes (n=3).

Table S4. Correlation coefficients between the new abstract principal components and the PAEs present in the bottled drinks.

variable	CAP1	CAP2	CAP3	CAP4	CAP5	CAP6
DMP	0.327	-0.102	0.19	-0.821	0.156	-0.072
DEP	0.147	-0.105	-0.128	-0.081	-0.092	-0.09
DIBP	0.358	0.283	0.363	0.222	-0.097	0.03
DBP	0.349	0.277	0.386	0.045	-0.058	0.029
BMEP	0.272	0.662	-0.625	-0.051	0.271	-0.01
DAP	0.28	-0.16	-0.273	-0.127	-0.428	-0.37
BEEP	0.333	-0.118	0.131	-0.028	0.271	0.095
BBP	0.345	-0.17	-0.195	0.147	-0.465	0.304
DCP	0.208	-0.042	-0.1	0.168	0.052	0.081
DHP	0.152	-0.141	-0.107	0.087	0.003	0.113
BMPP	0.255	-0.187	-0.059	0.024	-0.166	0.19
BBEP	0.247	-0.225	0.087	0.274	0.497	0.171
DEHP	0.051	0.316	0.315	0.158	-0.249	-0.341
DOP	0.131	-0.224	-0.108	0.167	0.118	-0.004
DNP	0.164	-0.241	-0.058	0.249	0.237	-0.738

.
